# High Temperature Oxidation Behavior of Creep Resistant Steels in Water Vapour Containing Environments

**DOI:** 10.3390/ma15020616

**Published:** 2022-01-14

**Authors:** Mária Hagarová, Gabriela Baranová, Martin Fujda, Miloš Matvija, Peter Horňak, Jozef Bednarčík, Daria Yudina

**Affiliations:** 1 Institute of Materials and Quality Engineering, Faculty of Materials, Metallurgy and Recycling, Technical University of Košice, 042 00 Košice, Slovakia; maria.hagarova@tuke.sk (M.H.); martin.fujda@tuke.sk (M.F.); milos.matvija@tuke.sk (M.M.); peter.hornak@tuke.sk (P.H.); 2 Institute of Physics, Faculty of Science, Pavol Jozef Šafárik University in Košice, 041 54 Košice, Slovakia; jozef.bednarcik@upjs.sk (J.B.); daria.yudina@student.upjs.sk (D.Y.)

**Keywords:** creep resistant steels, high temperature oxidation, water vapour, oxide layer, Cr oxide

## Abstract

This study describes the water vapour effect on the oxidation resistance of 9Cr creep resistant steels. Boiler P91 and MarBN steels were oxidized for 3000 h in a simulated humid atmosphere with ~10% water vapour. The oxidation kinetics had a stable course for 1000 h and was evaluated by the weight gain curves for both experimental steels and both oxidation temperatures. The oxidation rate was higher at 650 °C versus 600 °C, as reflected by the oxidation rate coefficient. A significant increase occurred after 1000 h of oxidation, which was related to the local breakdown oxide scale and oxide nodules were formed on steel. This oxidation behavior was influenced by the fact that a compact spinel structure of iron oxides and alloying elements were not formed on the steel. Analysis after 3000 h of exposure showed hematite Fe_2_O_3_ formed on the outer layer, magnetite Fe_3_O_4_ on the middle layer, and the bottom layer consisted of iron-chromium-spinel (Fe,Cr)_2_O_3_.

## 1. Introduction

Fossil fuel power plants remain an important source of energy production [1]. Due to environmental and economic factors, there is a demand for higher efficiency and more flexible operation of newly designed facilities. The application of ultracritical (USC) power plants, with significantly increased steam parameters, improves their efficiency, which reduces fuel consumption as well as CO_2_ emissions. Supercritical power plant generation with vapour cycle temperatures of 600–620 °C and pressures of up to 30 MPa involves the use of creep resistant steels for the production of superheater tubes, which determine the performance of the entire boiler plant vapour water cycle [2]. The next step is the development of steels for application at 650 °C. The new operating conditions are estimated to allow an efficiency up to 50% compared to 46% efficiency of the contemporary USC power plants [1].

Compared to austenitic heat-resistant stainless steels, 9Cr creep resistant steels are more cost-effective (due to nickel deficiency) [3]. At the same time, 9Cr creep resistant steels are characterized not only by high strength, but also by high thermal conductivity and low linear coefficient of expansion [4]. This fact significantly reduces their sensitivity to the applied thermal stress. In the development phase of 9Cr steels, the emphasis was primarily on improving their mechanical properties in the creep region [5] but when applied in operating conditions, it became obvious that a substantial part of the aggregates’ lifetime was mainly affected by their oxidation abilities [6,7,8].

Considering the nature of the heating system operation, the superheater tubes are exposed to high temperatures, which support the intensive oxidation process. A superheater tube failure can occur due to continuous exposition to high temperatures for a long time [9].

The kinetics and mechanism of oxide formation on the surface of the steel in the water vapour can differ significantly from the mechanism in oxygen or air. In 9–12Cr steels, where it relies on the formation of a chromia surface layer for protection, there is a faster degradation and breakaway of the initially formed protective Cr base oxide layer in steam. The detrimental effect of water vapour is a limiting factor for the application of the 9–12Cr steel.

After a certain period of oxidation, there is a formation of rapidly growing Fe_3_O_4_ and an inner scale consisting of Fe_3_O_4_ + (Fe,Cr)_3_O_4_. Fast ionic transport across the oxide layer results in vacancy transport. These vacancies can locally accumulate (or concentrate) in the oxide layer and/or the oxide/alloy interface. This process is accompanied by the breakaway of the initially formed protective Cr base oxide layer. Due to the presence of cavities, Fe transport is significantly reduced and hematite is formed at the interface with the environment (atmosphere). The effect of breakaway is often accompanied by the formation of nuclei (nodules) due to the formation of compressive oxide growth stresses and/or thermal stresses [10,11].

Long-term exposures carried out at temperatures of up to 650 °C showed that the oxidation resistance of 9Cr steels in air is excellent, due to the formation of a high-adhesion protective oxide layer. The main layer components are the oxides (Cr,Fe)_2_O_3_, Cr_2_O_3_ and Fe_2_O_3_ [12].

In the authors’ paper [7], the atmosphere containing 2–7 vol.% of water vapour, the oxide layer on the steel surface, formed at 550 °C, contained an outer layer composed of Fe_2_O_3_ and an inner two-phase layer of Fe_3_O_4_ and Fe_3_O_4_ + (Fe,Cr)_3_O_4_. Voids were observed in the outer layer. These were formed due to the loss of adhesion of the outer Fe_2_O_3_ layer to the Fe_3_O_4_ layer [13]. Through the voids in the oxide layer, oxygen transport occurred from the external environment. As the author states [7], in case of occurrence of these voids in Fe_3_O_4_, the value *p_O_*_2_ must be below the equilibrium value for the reaction 2 Fe_3_O_4_ + ½ O_2_ = 3 Fe_2_O_3_. The oxygen transport is very slow to ensure rapid layer growth through the aforementioned voids. In this case, the mechanism for stimulating oxide growth outside the voids does not work. The author of the paper [14] concluded that, in this case, oxygen transport is facilitated by the H_2_O presence. In this case, water vapour molecules also diffuse into the Fe oxide layer, thus allowing the oxygen transport for the reaction H_2_O = H_2_ + ½ O_2_. If the H_2_O_(g)_ transport is fast enough then its partial H_2_O pressure in the void approaches the external pressure of ~10^−2^ atm (~0.01 MPa). When local equilibrium between oxygen and oxide is reached, oxygen adsorption to the oxide layer takes place. If the adsorption occurs for the most part at the interface of the outer Fe_2_O_3_ layer and inner FeO.Cr_2_O_3_ layer, then the oxygen transport rate in the oxide layer promotes its growth. The penetration of water vapour molecules promotes enhanced oxidation and maintains high oxide growth rates with high Fe content and low protective efficiency [7,15].

The application temperature of 9Cr creep resistant steels in high temperature applications is limited by the breakaway of Cr-rich oxides due to the presence of higher volume of water vapour in the operating environment.

This study focuses on the oxidation kinetics, morphology, and structure of the oxide layer over the cross-section and its chemical analysis. The cause of the increase in oxidation rate was discussed, the course and the mechanism of changes in the chemical composition of the oxide layer after 1000 h of exposure were observed. The ability to diffuse and form a compact protective inner oxide layer based on oxides of substitution elements, not only Cr but also minor alloying additions Mn, Mo, Si, and Co was a condition for maintaining antioxidant resistance at the tested temperatures of 600 and 650 °C.

## 2. Materials and Methods

In this study, two 9Cr creep resistant steels, P91 and MarBN, were examined. Their chemical compositions are shown in Table 1 and Table 2. P91 steel consists of a fine polyhedral ferrite matrix with precipitates. There are two kinds of precipitates: M23C6 (M = Cr, Fe, Mo) and finely dispersed MX-type carbides. The structure of 9Cr steel MarBN is martensitic-ferritic which contains M23C6 (M = Fe, Cr, Mo, W) carbides and MX type carbonitrides.

Samples for high temperature oxidation tests were cut from thick-walled tubes: from P91 steel with a wall thickness of 12.21 mm and from MarBN steel with a wall thickness of 12.27 mm, with a small 2 mm diameter hole near one edge. Each specimen was cleaned with ethanol immediately prior to oxidation testing.

The isothermal oxidation method was used to study the high-temperature oxidation of two tested steels. The oxidation rate was determined by weighing at specified time intervals (before, during and after the oxidation process).

Oxidation tests were carried out in a resistance heating furnace (ATEKO, Hradec Králové, Czech Republic). A simulated humid atmosphere with ~10% water vapour was created by dosing distilled water onto a horizontal retort in the furnace chamber. The flow rate of the peristaltic pump was adjusted according to the defined water vapour content of the test atmosphere. The air with water vapour content represented an environment that originates during fossil fuels combustion. Water vapour is considered to be the main degrading component [7,16,17].

The isothermal oxidation method was used for this experiment and the steel oxidation rate was determined by discontinuous weighing.

The oxidation temperatures were 600 and 650 °C, the oxidation times at each temperature were 100, 250, 500, 750, 1000, 2000, and 3000 h respectively. Two parallel specimens were used for each temperature and time.

Weight measurements were carried out before and after the individual oxidation periods on analytical scales KERN ALT 220-5DAM (KERN & SOHN, Balingen, Germany) with the accuracy of 0.0001 g. After the oxidation test, the oxidation weight gain was analyzed. The oxide layer documentation on the surface and measurement of its thickness were carried out by the OLYMPUS VANOX-T light microscope (Olympus, Tokyo, Japan). The morphology and chemical composition of the specimen oxidized surface were examined by the scanning electron microscope JEOL JSM 7000F (JEOL, Tokyo, Japan) included into the energy dispersive spectroscopy (EDS). The phase identification of the oxide film surface was determined by X-ray diffraction (XRD) experiments in reflection mode with Bragg-Brentano parafocusing geometry using a Rigaku Ultima IV multipurpose diffractometer (Rigaku, Tokyo, Japan). X-ray lamp with Cu-Kα_1,2_ radiation (λ = 0.154 nm) was used.

## 3. Results and Discussion

### 3.1. Oxidation Dynamic Curves

**Figure 1:**
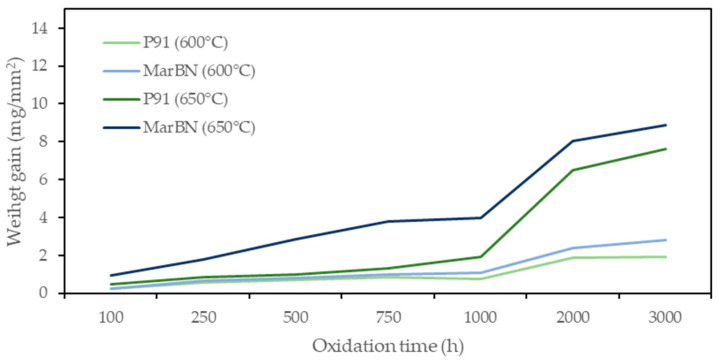
Oxidation weight gain curves of P91 and MarBN steels in a simulated humid atmosphere with ~10% water vapour at 600 °C and 650 °C.

The oxide weight gain curves of oxide samples under different oxidation conditions (temperature, time) are shown in Figure 1. Oxidation rate refers to the mass gain of the samples in unit area and unit time, which can be used to characterize the oxidation intensity of materials [18].

**Figure 2:**
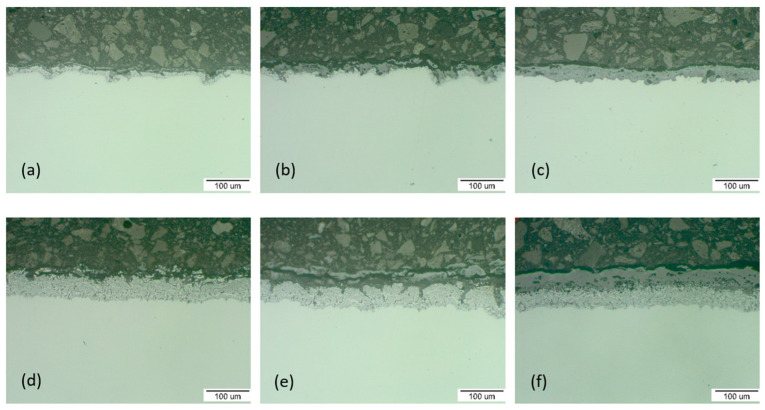
P91 cross-section analysis for: (**a**) 100 h; thickness of the oxide layer 18.97 µm, (**b**) 1000 h; 23.05 µm, (**c**) 3000 h; 49.37 µm and MarBN for: (**d**) 100 h; 20.64 µm, (**e**) 1000 h; 26.93 µm (**f**) 3000 h; 57.91 µm oxidized at 600 °C.

**Figure 3:**
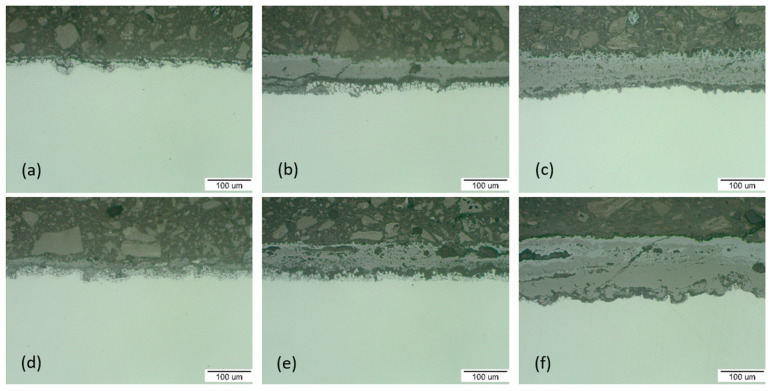
P91 cross-section analysis for: (**a**) 100 h; thickness of the oxide layer 26.93 µm, (**b**) 1000 h; 37.10 µm, (**c**) 3000 h; 107.70 µm and MarBN for: (**d**) 100 h; 34.28 µm, (**e**) 1000 h; 70.71 µm, (**f**) 3000 h; 124.17 µm oxidized at 650 °C.

The weight gain for P91 and MarBN steels increased with increasing oxidation temperature and oxidation time. As can be seen from the oxidation curves in Figure 1, the oxidation of experimental steels was stable during the first 1000 h. During 1000 h of oxidation, a stable weight gain was observed, which can be explained by a thin protective oxide layer formation on the steel surface without its significant breakaway, Figure 2 and Figure 3. It is assumed that in this time interval, the surface of the steel was mainly covered by Cr-rich oxides, which could prevent the penetration of oxygen, and thus no significant subsurface oxidation occurred [13].

This phenomenon indicates that P91 steel had a better antioxidant effect at both 600 °C and 650 °C. Steels with a ferrite content or ferritic steels often show increasing oxidation resistance with increasing oxidation temperature [19,20].

The analysis of different steel oxidation behavior in water vapour is a tool for better understanding of the oxidation mechanism and leads to the following conclusions, to which several authors have come in their works [7,14,21]: the rate-limiting step of the whole corrosion process is the outward iron migration to form the outer layer and iron migrates when the ions increase the oxygen potential towards high oxygen potential that is present on the outer surface. In this process, the ions oxidize from Fe^2+^ to Fe^3+^. This leads to a lower Fe^2+^/Fe^3+^ ratio on the outer layer surface versus the oxide-steel interface. The compact oxide film formation on the surface prevents contact between the steel surface and the furnace area environment. Since the oxidation rate is controlled by diffusion, the mass gain is also lower [21].

After changing the oxidation rate and especially the last 1000 h of the process (in the time interval of 1000–2000 h), the effect of long-term higher oxidation temperature was significantly manifested, and the oxidation mass gain reached the highest value. After 2000 h, a slowdown in the oxidation rate was observed for all tested steels and temperature conditions. The authors in [22] described that after the breakaway in the oxide layer, there was a certain re-passivation, and the result was an overlap of nodules in the continuous thick oxide scale. This effect was reflected in a slowing down of the oxidation rate.

### 3.2. Microscopic Analysis

The cross-sectional morphologies of the two steels after oxidation for 100 h, 1000 h and 3000 h at 600 °C and 650 °C were observed, as shown in Figure 2 and Figure 3. It was shown that the material oxidation of up to 1000 h was not significant and the oxide layers were relatively thin. The oxide layer covered the steel surface and in the form of protrusions it interfered with the steel material.

The oxide layer thickness measurement was carried out on a light microscope. The measured values, shown in Figure 2 and Figure 3, were obtained as the average of 10 values of measurements on the documented surfaces. The trend of oxide layer thickness growth followed the trend of mass growth. At the beginning of the oxidation at 600 °C (after 100 h), a thin protective layer of oxides with a thickness of 18.97 µm for P91 steel and 20.64 µm for MarBN was formed. At the end of the oxidation process (after 3000 h), the oxide layer thickness was 49.37 µm for P91 and 57.91 µm for MarBN steel.

At the beginning of the oxidation at 650 °C (after 100 h), the protective oxide layer thickness was 26.93 µm for P91 and 34.28 µm for MarBN. At the end of the oxidation process (after 3000 h), the oxide layer thickness was 107.70 µm for P91 and 124.17 µm for MarBN steel. After 1000 h of P91 steel and MarBN steel oxidation at both exposure temperatures, cracks and Fe-oxide rich nodules were formed in the Cr-depleted subsurface area. The presence of water vapour helped with the formation of volatile chromium-containing species, thus increasing the kinetics of chromium depletion in the metal subsurface location [14,22]. Another course indicated a slowdown in the growth of the oxide layer, which could be caused by surface re-passivation, when the breakaways were covered with an oxide layer rich in Fe, but also Cr and minor alloying additives [23].

### 3.3. Morphology and Composition of Oxide Layers after Oxidation

**Figure 4:**
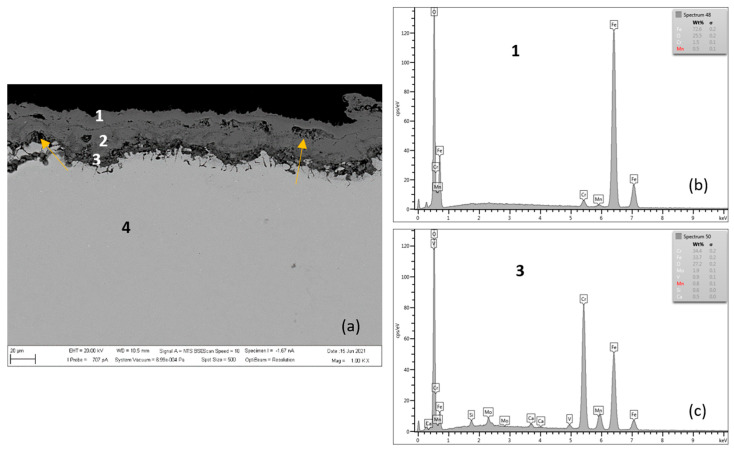
P91 after 2000 h at 600 °C (**a**), EDX analysis from location 1 (**b**) (outer oxide layer), and location 3 (**c**) (interface substrate/oxide layer).

**Figure 5:**
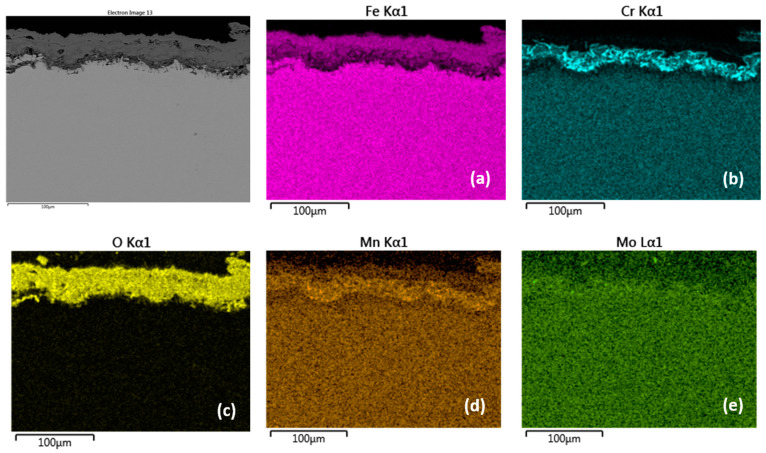
Cross-sectional SEM morphologies and EDS spectra analysis (**a**–**e**) of oxide films after oxidation for 2000 h, steel P91 at 600 °C.

**Figure 6:**
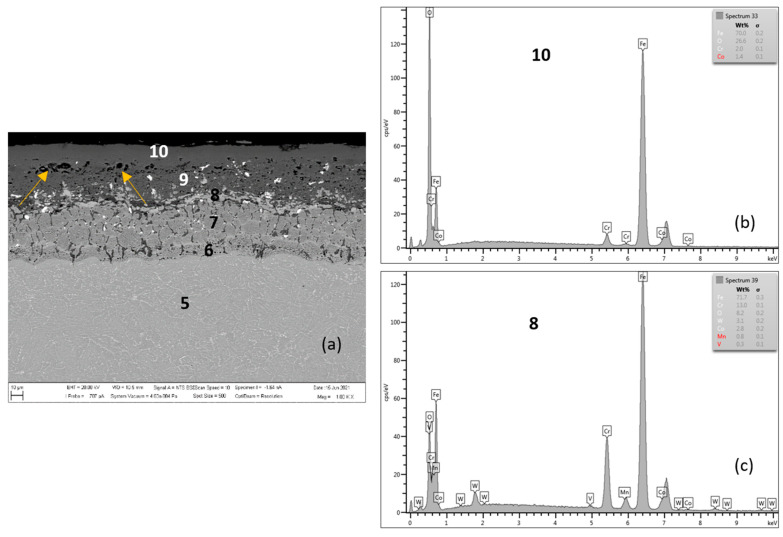
MarBN after 2000 h at 600 °C (**a**), EDX analysis from location 10 (**b**) (outer oxide layer) and location 8 (**c**) (interface substrate/oxide layer).

**Figure 7:**
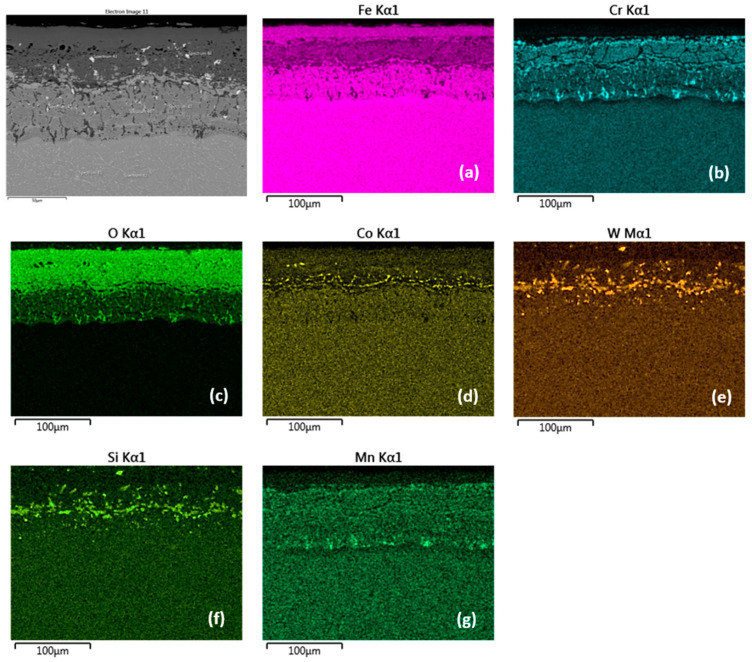
Cross-sectional SEM morphologies and EDS spectra analysis (**a**–**g**) of oxide films after oxidation for 2000 h, steel MarBN at 600 °C.

Figure 4, Figure 5, Figure 6 and Figure 7 and Table 3 and Table 4 present the qualitative and quantitative cross-section analysis for the tested P91 and MarBN steels after 2000 h oxidation at 600 °C. This oxidation time is interesting because of the more significant change in the oxidation rate as shown in Figure 1. The oxide layer morphology on P91 steel is documented in Figure 4. The average oxide layer thickness was 35.74 µm. The results of the quantitative EDX analysis, shown in Table 3, indicated a high 34.4 wt.% Cr at the steel/oxide layer interface (location 3 in Figure 4 and in Table 3), and also in the inner layer of 14.5 wt.% (location 2 in Figure 4 and in Table 3). The Cr-rich layer at the interface was locally discontinuous at locations with lower Cr contents, as illustrated by the EDS map in Figure 5b. As reported by the authors in [24], 28 at.% Cr corresponds to the maximum chromium content in spinel oxide, which is equivalent to the FeCr_2_O_4_ stoichiometry. The inner layer edge was accompanied by the formation of protrusions, which were covered with Fe oxides, as confirmed by Figure 5c. In the oxide layer cross section, a location is visible that separates the lower part from the upper part of the oxides. The paper [24] describes that this is an interface that separates the bottom spinel oxide from the outer oxide layer area. As the authors of the paper further state, the above interface corresponds to the original sample surface. That is, oxide layer regions were formed by diffusion of metal cations towards the outer surface, while the layer below the interface was enlarged by oxygen transport inside the material. Thus, it can be concluded that the formed magnetite and hematite layers, after their loss of compactness in a moisture-containing environment, grew by the iron diffusion in the outward direction. The cross-sectional distribution of elements after 2000 h shows that the oxidation process was in oxide film formation stage which means that the alloy elements diffused outwards from the steel and the oxygen inwards, Figure 5a–e. Mn and Mo were rather located in the inner diffusion zone part. Isolated islands of oxide nodules (indicated by arrows in Figure 4) were also part of the layer, which were the result of the water vapour based environment acting on the steel surface [21,24].

The further growth of the layer largely depends on the transport process within the nodules.

Because iron transport is significantly reduced due to the presence of cavities, hematite is formed at the interface with the oxidizing environment of the furnace (location 1 in Figure 4 and Table 3). Depending on the oxidation conditions, “depassivation” processes can occur inside the oxide layer or partial peeling of the oxide layers [22,25].

Figure 6 shows the oxidized surface of MarBN steel after 2000 h of oxidation at 600 °C. The average oxide layer thickness was 62.86 µm. The quantitative EDX analysis results, shown in Table 4, showed a high Cr content of 13.0 wt.% at the steel/oxide layer interface (location 8 in Figure 6 and in Table 4), and also in the inner oxide layer at 14.4 wt.% (location 9 in Figure 6 and in Table 4). The Cr content indicated in locations 6 and 7 was part of the steel substrate chemical composition. As documented in Figure 6, the outer oxide layer was interrupted by nodules (indicated by arrows in the figure), which are formed as the result of the water vapour action in the oxidizing environment on the steel surface [21,24].

Compared to the oxide layer on MarBN steels (Figure 6 and Figure 7), the distribution of Cr and Mn in the oxide layer on P91 steels is uniform (Figure 4 and Figure 5), although local discontinuities were observed. The lower diffusion activity in the martensitic-bainitic steel MarBN led to a less continuous distribution of Cr and Mn in the inner oxide layer. For the reason mentioned above, the antioxidant effect of the surface of MarBN steel was lower than that of P91 steel with a ferritic matrix [20,26]. It was reflected in the larger average oxide layer thickness of 62.86 µm compared to the average oxide layer thickness of 35.74 µm on P91 steel.

This assumption was also confirmed by the EDS map of element distribution that diffused in the forming Fe oxide layer during the oxidation process, Figure 7a–g. The Cr distribution shows a higher Cr content at the substrate/layer interface and in the inner layer, Figure 7b. However, this region is locally interrupted by locations with lower Cr content. The outer layer surface contains Cr, however, the EDX analysis showed a low 1.5 wt.% Cr content (location 10 in Table 4). A compact Cr-enriched layer with Cr content of at least 7.0 wt.% is required for a sufficiently effective oxide layer [7,17]. The cross-sectional distribution of elements further showed that Co, W and Si were more a part of the inner oxide layer (Figure 7d–f) in contrast to Mn, whose distribution in the layer is uniform (Figure 7g).

According to [22,27], the chromium content is essential for these steels, but even small amounts of additional alloying elements (e.g., Mn, Mo, Si) can have a significant effect on the oxidation behavior of steel by influencing either the nucleation behavior of the protective chromium layer (and thus the behavior of cracks in healing and by this also the healing behavior of cracks) or the behavior of chromium removal in the subsurface zone of steel during oxidation or also the chromium depletion behavior of the steel subsurface location during oxidation.

**Figure 8:**
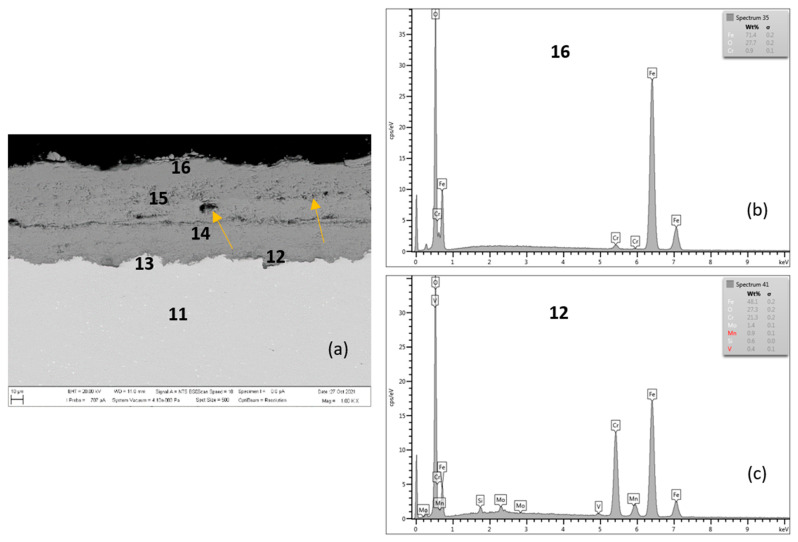
Steel P91 after 2000 h at 650 °C (**a**), EDX analysis from location 16 (**b**) (outer oxide layer) and location 12 (**c**) (interface substrate/oxide layer).

**Figure 9:**
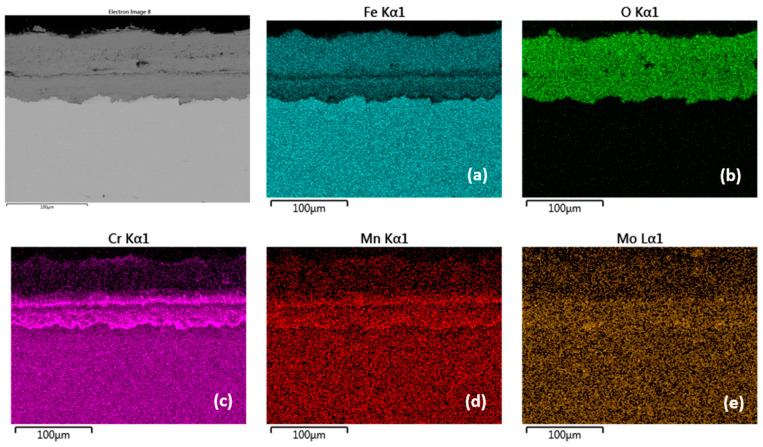
Cross-sectional SEM morphologies and EDS spectra analysis (**a**–**e**) of oxide films after oxidation for 2000 h, steel P91 at 650 °C.

**Figure 10:**
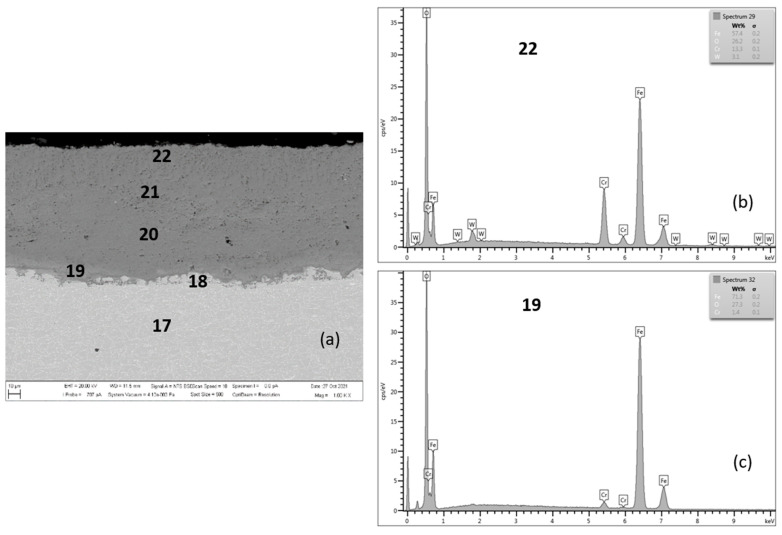
MarBN after 2000 h at 650 °C (**a**), EDX analysis from location 22 (**b**) (outer oxide layer) and location 19 (**c**) (interface substrate/oxide layer).

**Figure 11:**
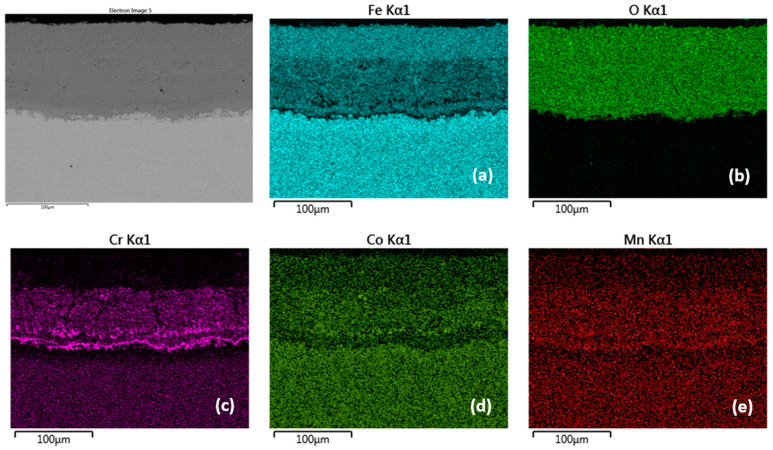
Cross-sectional SEM morphologies and EDS spectra analysis (**a**–**e**) of oxide films after oxidation for 2000 h, steel MarBN at 650 °C.

In Figure 8, Figure 9, Figure 10 and Figure 11 and Table 5 and Table 6, the qualitative and quantitative cross-section analysis for the tested P91 and MarBN steels after 2000 h oxidation at 650 °C is documented.

Figure 8 shows the P91 steel after 2000 h oxidation at 650 °C. The average oxide layer thickness was 76.52 µm. The quantitative EDX analysis results, shown in Table 5, showed a high Cr content of 21.3 wt.% at the steel/inner oxide layer interface (location 12 in Figure 8 and in Table 5), and also in the inner layer with a Cr content of 11.7 wt.% (location 14 in Figure 8 and in Table 5). The 9.6 wt.% Cr shown in area 11, or 13, was part of the steel substrate chemical composition. The outer oxide layer (locations 15 and 16) had Cr contents at the level of tenths of 0.7–0.9 wt.%. As documented in Figure 8, the outer oxide layer was interrupted by heterogeneities in its structure (indicated by arrows in the figure), which were formed as the result of the water vapour acting in the oxidizing environment on the steel surface [21,24].

Non-protective oxide layers (locations 15 and 16 in Figure 8) formed on the surface, composed of an outwards grown Fe_2_O_3_ with a Cr depleted steel subsurface localization. As the conclusions of the research [17] state, this process leads to the local formation of fast-growing iron-rich oxide nodules, and this surface was completely covered with a non-protective-oxide scale. As further stated by the authors of the work [17], with the growing oxidation time, fast-growing nodules start to cover large parts of the surface and grow together, finally ending up in the continuous thick oxide scale. This fact was confirmed even after 2000 h oxidation at 650 °C steel P91, when the average thickness of the outer non-protective oxide layer (Figure 8 and Figure 9) increased significantly compared to the outer oxide layer after 2000 h oxidation at 600 °C (Figure 5 and Figure 6).

There is a visible interface in the oxide layer structure along the cross-section (similar to the oxide layer of P91 steel oxidized for 2000 h at 600 °C). This interface separates higher Cr content regions from the outer oxide parts with lower Cr content. The EDS map shows the distribution of elements that diffused in the forming Fe oxide layer during the oxidation process, Figure 9a–e. The Cr distribution shows a higher Cr content at the substrate/layer interface, and at the interface between the lower and upper parts of the oxide layer, Figure 9c. On the outer surface of the oxide layer, the Cr content is significantly lower, as confirmed by EDX analysis (location 16 in Figure 8 and Table 5). The fact that chromium is present in higher amounts only inside the growing layer can be explained by lower diffusivity of Cr^3+^ versus Fe^2+^ in oxides [28]. For P91, the outward growing layer is composed of chromium-bearing hematite, while the inward growing oxide reached a composition with higher Cr content in some areas. The cross-sectional distribution of elements further showed that Mn and Mo were more part of the inner oxide layer, Figure 9d,e.

Figure 10 documents a cross-section of MarBN steel after 2000 h of oxidation at 650 °C. The average oxide layer thickness was 97.75 µm. The results of the quantitative EDX analysis, shown in Table 6, showed a high Cr content of 15.2 wt.% at the steel/inner oxide layer interface (location 19 in Figure 10 and Table 6), and also in the inner layer (location 20 in Figure 10 and Table 6) and at the inner/outer oxide layer interface (location 21 in Figure 10 and Table 6). The Cr content at locations 18 and 19 is part of the steel chemical composition. There is an interface in the structure of the oxide layer after the cross-section with different Cr and Fe contents in the inner oxide layer, versus the outer oxide layer, as shown in Table 6 according to Figure 10. This observation is also confirmed by the EDS map for Fe and Cr in Figure 11a,c. Such an interface was formed in the layers of all tested steels, only with different “sharpness” (Figure 4, Figure 6, Figure 8 and Figure 10). The EDS map with distribution of elements that diffused in the forming Fe oxide layer during the oxidation process, Figure 11a–e, shows at the same time a higher concentration of Co and Mn in the inner oxide layer versus the outer one. This may be due to the lower diffusivity of the above elements from steel, compared to Fe [28].

The growth of the average thickness of the oxide layer was mainly supported by the formation of non-protective behavior “low-Cr” oxides Fe_2_O_3_. This process occurs when the kinetics of chromium subsurface location depletion exceeds the formation of a layer with a protective antioxidant effect [17].

### 3.4. Surface Morphology of the Oxide Film after 3000 h Oxidation at 600 °C

**Figure 12:**
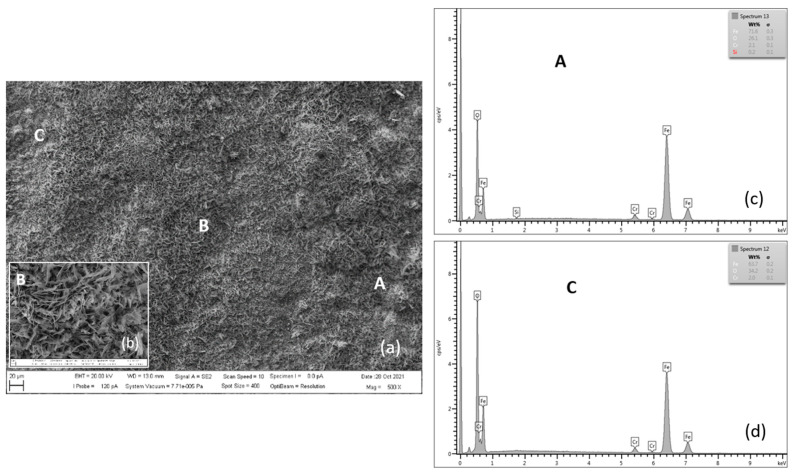
SEM morphology of the P91 steel surface layer oxidized for 3000 h at 600 °C (**a**), with B location detail (**b**), EDX energy spectrum of steel oxide surface (**c**,**d**).

**Figure 13:**
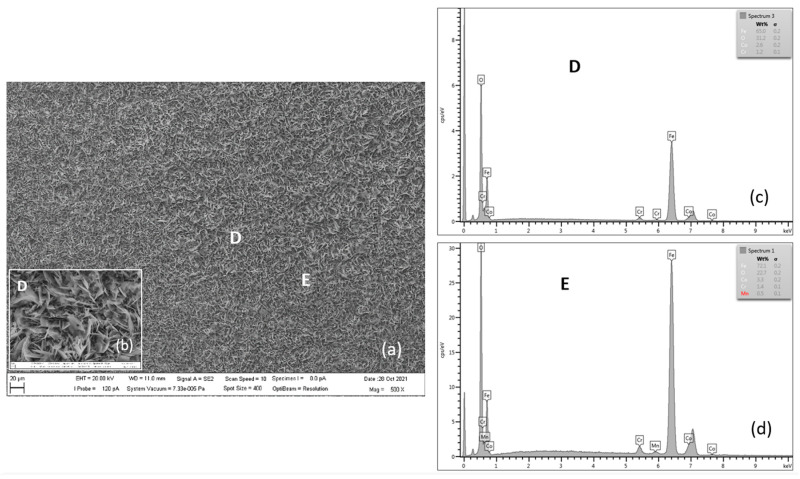
SEM morphology of surface layer of MarBN steel oxidized for 3000 h at 600 °C (**a**), with D location detail (**b**), EDX energy spectrum of steel oxide surface (**c**,**d**).

**Figure 14:**
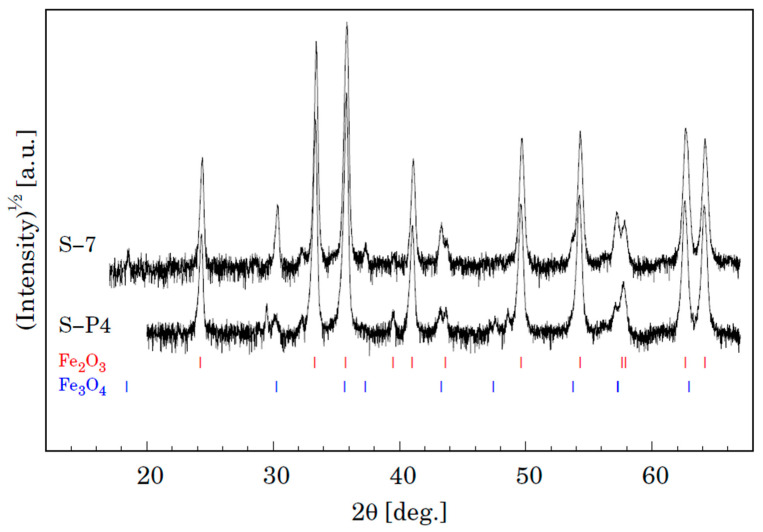
XRD patterns for samples S-P4 (P91) and S-7 (MarBN) oxidized for 3000 h at 600 °C. Vertical lines shown at the bottom depict positions of Bragg reflections belonging to Fe_2_O_3_ and Fe_3_O_4_.

**Figure 15:**
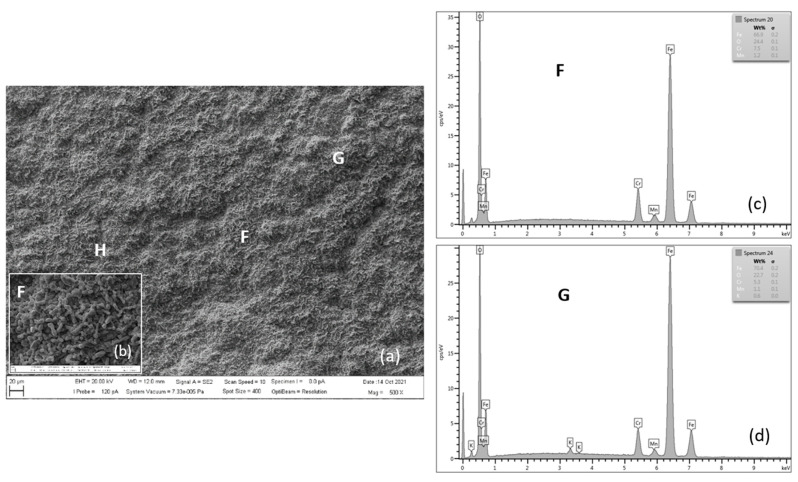
SEM morphology of surface layer of P91 steel oxidized for 3000 h at 650 °C (**a**), with F location detail (**b**), EDX energy spectrum of steel oxide surface (**c**,**d**).

Figure 12, Figure 13, Figure 14 and Figure 15 document the steel surface morphology after continuous oxidation of 3000 h. Figure 12 shows the oxidized surface of P91 steel after 3000 h of oxidation at 600 °C. As shown in the above figure, the surface morphology is formed by acicular oxides (locations A and B) and nodular oxides (location C).

According to the authors [8], acicular oxide fills the voids between the spinel oxides and the surface becomes more compact, which results in improvement in the antioxidant capacity of materials. In case, after a certain period of oxidation, the healing of the steel surface occurs as the authors [22,26,27] have also considered, the course of the oxidation process will slow down. During the research of our tested steels, there was a slight slowdown in oxidation after 2000 h.

Surface nodular oxides are often separated by voids and cracks, which can lead to the oxide region separation [10]. Detail from location B shows the occurrence of blade-shaped crystals on the scale surface. As reported by the authors in the paper [24] the formation of blade-shaped hematite crystallites at the scale surface in wet O_2_ was also observed for pure Fe.

Table 7 shows the quantitative EDX analysis results of the experimental P91 steel oxide surface after 3000 h of oxidation at 600 °C. The main elements on the tested steel surface are Fe, O, Cr, Mn and Si.

Figure 13 documents the oxide layer morphology on the surface of MarBN steel after 3000 h of oxidation at 600 °C. A relatively uniform oxide was formed on the surface. The main effect of the water vapour presence is a somewhat higher mass gain than in dry O_2_ and the appearance of blade-shaped crystals on the scale surface [24]. D location detail shows the above type of hematite crystallites, similar to those on the P91 steel in Figure 12.

Results of quantitative analysis in Table 8 show the occurrence of the elements: Fe, O, Co, Cr, and Mn. Elements noted above are part of the oxide layer.

The XRD analyses of the samples also provide important information on the oxidation phenomena [29]. Phase analyses of the surface oxide formed on P91 and MarBN steels by XRD after oxidation exposed in humid atmosphere with ~10% water vapour at 600 °C for 3000 h are shown in Figure 14. It is evident that both samples show the presence of two iron oxides, that is, Fe_2_O_3_ (trigonal, space group R-3c (167) and Fe_3_O_4_ (cubic, space group Fd-3 m (227). Identification of some peaks was not possible due to minor phases in the oxide layer. We can assume the presence of Cr and Co oxides, as shown by the results of the quantitative analysis in Table 7 for steel P91 and Table 8 for MarBN steel.

### 3.5. Surface Morphology of the Oxide Film after 3000 h Oxidation at 650 °C

9Cr steels may show increasing oxidation resistance with increasing temperature. Higher diffusion rates of the elements forming the protective oxide layers, such as Cr, Mn, and Co, lead to more extensive incorporation of these elements into the oxide layer, thus improving their protective effect [22,27].

Figure 15 shows the oxidized surface of P91 steel for 3000 h of oxidation at 650 °C. The surface morphology of the oxide layer on steel is formed by nodular-like (locations H and G) and spinel-like forms (location F). According to the authors of [8], surface nodular oxides are often separated by cavities and cracks, which can lead to the separation of the oxide region [10]. Detail from location F shows the occurrence of iron-chromium-spinel oxides.

Table 9 shows the quantitative EDX analysis results of the experimental P91 steel oxide surface after 3000 h of oxidation at 650 °C. The main elements on the tested steel surface are Fe, O, Cr and Mn.

At this stage, breakaway oxidation has occurred, resulting in a very rough scale with hematite blades and nodules (Figure 15) [21]. The presence of a compact layer (film) of spinel oxides can better prevent further oxidation inside the steel [8].

**Figure 16:**
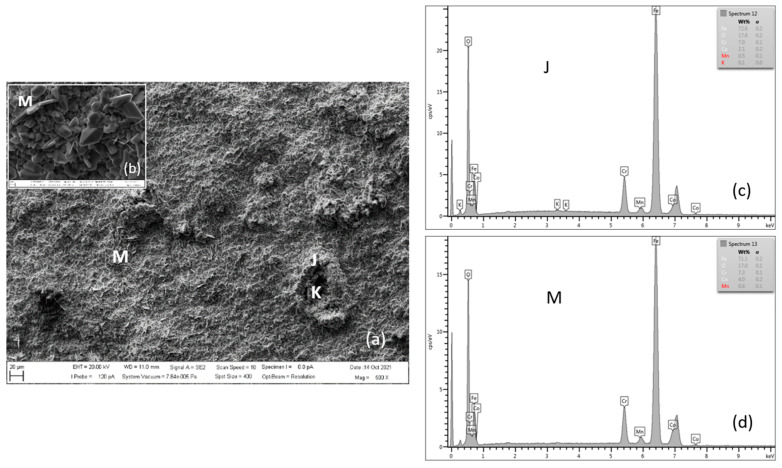
SEM morphology of surface layer of MarBN steel oxidized for 3000 h at 650 °C (**a**), with M location detail (**b**), EDX energy spectrum of steel oxide surface (**c**,**d**).

Figure 16 shows the surface morphology of the oxide layer on MarBN steel after 3000 h of oxidation at 650 °C. The surface morphology is more striking compared to the surface of P91 steel. On the other hand, it is more homogeneous in terms of Cr content. Iron-chromium-spinel oxides form with more than 7 wt.% can be seen in detail from location M (Table 10, location M). According to the authors of [17], the change in oxidation resistance between protective and non-protective behavior occurs at a content of about 7% Cr for 9–12% Cr steel.

**Figure 17:**
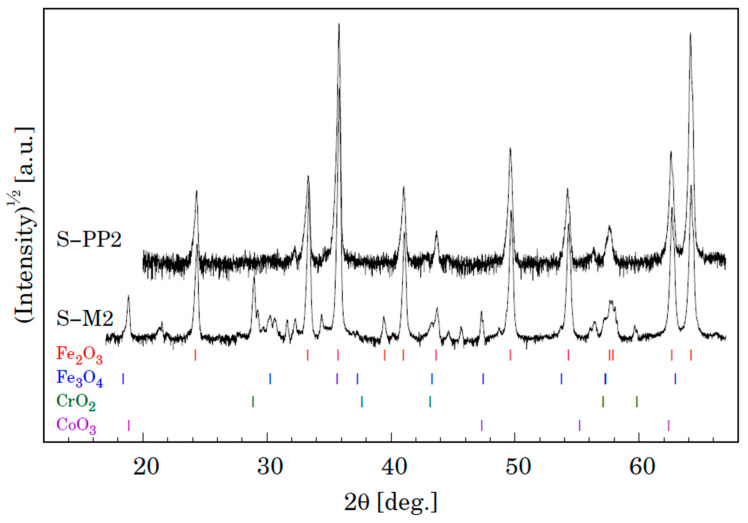
XRD patterns for samples S-P4 (P91) and S-7 (MarBN) oxidized for 3000 h at 650 °C. Vertical lines shown at the bottom depict positions of Bragg reflections belonging to Fe_2_O_3_ and Fe_3_O_4_.

Phase analysis of the surface oxide formed on P91 and MarBN steels by XRD after oxidation for 3000 h at 650 °C is shown in Figure 17. Both samples show the presence of two iron oxides, that is, Fe_2_O_3_ and Fe_3_O_4_, as in the case of steels P91 and MarBN oxidized for 3000 h at 600 °C in Figure 14. As documented in Figure 17, in contrast to the above-mentioned steels, in this case, Cr and Co oxides were also identified. Identification of Cr oxide with Cr content above 7 wt.% corresponds to iron-chromium-spinel oxides. Identification of some peaks was not possible due to minor phases in the oxide layer.

## 4. Conclusions

This study investigated the oxidation behavior of 9Cr creep resistant steels in a simulated humid atmosphere with ~10 vol.% water vapour at 600 and 650 °C for 3000 h. The main result of this paper was a correlation of the evolving microstructure with the growth kinetics of the oxide layer. The conclusions are as follows:

1. The experimental steels P91 and MarBN had similar oxidation kinetics at 600 and 650 °C. At 650 °C, a higher oxidation rate was found for both steels. For 1000 h, the oxidation process had a stable course. After 1000 h of oxidation, there was an increase in the oxidation rate, which was related to the oxide nodules formation in the forming outer oxide layer. The occurrence of oxide nodules is the result of the water vapour action on a steel surface. A slight decrease in the oxidation rate after 2000 h indicated a probable “healing process” of the steel surface. The enhanced diffusion at higher temperatures and longer oxidation times allows the surface coverage (with nodules present) by a growing layer of Fe oxide with the presence of Cr and minor alloying elements, which increased the oxidation resistance of the surface;
2. The oxides’ thickness on the P91 and MarBN steel surface increased as the oxidation temperature and oxidation time increased. After continuous oxidation for 1000 h with a stable course, the average oxide layer thickness had the following values: at the oxidation temperature of 600 °C, 23.05 μm for P91 steel and 26.93 μm for MarBN steel; at the oxidation temperature of 650 °C, it was 37.10 μm and 70.71 μm for P91 and MarBN steel, respectively. After 3000 h of oxidation, the increase in thickness was recorded as follows: at the oxidation temperature of 600 °C to 49.37 μm for P91 and 57.91 μm for MarBN steel; at 650 °C the increase was 107.70 μm for P91 steel and 124.17 μm for MarBN steel;
3. The cross-section analysis showed that the oxide layer on both documented steels was formed by three regions: the steel substrate/oxide interface with higher wt.% Cr, which corresponded to (Fe,Cr)_3_O_4_ spinel oxide, the inner Fe_3_O_4_ magnetite oxide layer, which included spinel based on (Fe,Cr)_3_O_4_, or (Mn,Fe,Cr)_3_O_4_, and the outer oxide layer formed by Fe_2_O_3_ hematite with a low Cr content;
4. The effect of the mixed atmosphere of air and water vapour was manifested by the formation of discontinuities in the spinel oxide structure at the steel/inner oxide layer interface and the formation of oxide nodules in the inner and outer oxide layers;
5. The microscopic and XRD analysis of the oxide layer surface after a total 3000 h oxidation showed the morphology heterogeneity with the presence of blade-shaped hematite crystallites, acicular and nodular oxides, and spinel-like forms. Iron-chromium-spinel oxides with more than 7 wt.% Cr in a compact layer provides significant antioxidant protection to steels.

The application of the findings of this study can lead to modifications of the metallurgy of steel production, including chemical composition, as well as the choice of the optimal exposure temperature. Equally important is the functional life (respectively residual life) estimate of devices made of studied 9Cr steels.

## Figures and Tables

**Table 1:** Chemical composition of P91 steel.

Element	C	Mn	Si	P	S	Cr	Ni	Mo	B	N	V
min (wt.%)	0.08	0.30	0.20	-	-	8.00	-	0.85	0.0015	0.03	0.18
max (wt.%)	0.12	0.60	0.50	0.02	0.01	9.50	0.4	1.05	0.0070	0.07	0.25

**Table 2:** Chemical composition of of MarBN steel.

Element	C	Mn	Si	P	S	Cr	Ni	Mo	W	Co	B	N
min (wt.%)	0.06	0.40	0.20	-	-	8.00	-	-	2.50	2.80	0.01	0.005
max (wt.%)	0.10	0.50	0.35	0.020	0.008	9.00	0.15	0.10	3.00	3.20	0.015	0.015

**Table 3:** Quantitative EDX analysis, sample P91 oxidized at 600 °C for 2000 h (in wt.%).

Element	Location 4	Location 3	Location 2	Location 1
Fe	89.2	33.7	57.0	72.6
O	-	27.2	25.9	25.5
Cr	9.5	34.4	14.5	1.5
Mn	-	0.8	0.7	0.5
Mo	1.0	1.9	1.1	-
V	-	0.9	0.4	-
Si	0.3	0.6	0.4	-

**Table 4:** Quantitative EDX analysis, MarBN sample oxidized at 600 °C for 2000 h (in wt.%).

Element	Location 5	Location 6	Location 7	Location 8	Location 9	Location 10
Fe	83.5	71.7	72.4	71.7	54.7	70.0
O	-	8.2	8.8	8.2	25.4	26.6
Cr	9.2	13.0	9.8	13.0	14.4	2.0
Co	3.4	2.8	3.2	2.8	3.0	1.4
W	3.3	3.1	5.2	3.1	2.0	-
Mn	0.6	0.8	0.4	0.8	-	-
Si	0.2	2.6	0.3	0.2	-	-
V	-	0.3	0.2	0.3	0.4	-

**Table 5:** Quantitative EDX analysis, P91 steel oxidized at 650 °C for 2000 h (in wt.%).

Element	Location 11	Location 12	Location 13	Location 14	Location 15	Location 16
Fe	88.1	48.1	93.1	59.1	73.1	71.4
O	-	27.3	-	25.8	26.2	27.7
Cr	9.6	21.3	5.3	11.7	0.7	0.9
Mn	0.4	0.9	0.2	-	-	-
Mo	1.0	1.4	1.0	1.8	-	-
V	0.2	0.4	0.2	0.3	-	-
Si	0.3	0.6	0.2	0.5	-	-

**Table 6:** Quantitative EDX analysis, sample MarBN oxidized for 2000 h at 650 °C (in wt.%).

Element	Location 17	Location 18	Location 19	Location 20	Location 21	Location 22
Fe	83.5	79.6	54.5	52.4	54.8	70.2
O	-	5.4	26.5	26.8	27.0	27.5
Cr	9.2	6.3	15.2	14.7	12.4	1.8
Co	3.2	4.9	-	4.2	3.6	-
W	3.4	3.9	3.4	1.4	1.7	0.5
Mn	0.5	-	0.3	0.5	0.4	-
V	0.2	-	0.2	-	0.2	-

**Table 7:** Quantitative EDX analysis, sample P91 oxidized for 3000 h at 600 °C (in wt.%).

Element	Location A	Location B	Location C
Fe	63.7	71.6	72.2
O	34.2	26.1	24.6
Cr	2.0	2.1	2.3
Mn	-	-	0.3
Si	-	0.2	0.6

**Table 8:** Quantitative EDX analysis, sample MarBN oxidized for 3000 h at 600 °C (in wt.%).

Element	Location D	Location E
Fe	65.0	72.1
O	31.2	22.7
Co	2.6	3.3
Cr	1.2	1.4
Mn	-	0.5

**Table 9:** Quantitative EDX analysis, sample P91 oxidized for 3000 h at 650 °C (in wt.%).

Element	Location F	Location G	Location H
Fe	66.9	70.4	68.5
O	24.4	22.7	23.9
Cr	7.5	5.3	6.2
Mn	1.2	1.1	1.4

**Table 10:** Quantitative EDX analysis, sample MarBN oxidized for 3000 h at 650 °C (in wt.%).

Element	Location J	Location K	Location M
Fe	72.6	68.6	71.1
O	17.6	21.1	17.0
Cr	7.0	7.2	7.3
Co	2.1	2.8	4.0
Mn	0.5	0.3	0.6

## Data Availability

The data presented in this study are available upon request from the first author.
